# Moving to Aqueous Binder: A Valid Approach to Achieving High‐Rate Capability and Long‐Term Durability for Sodium‐Ion Battery

**DOI:** 10.1002/advs.201700768

**Published:** 2018-01-20

**Authors:** Jing Zhao, Xu Yang, Ye Yao, Yu Gao, Yongming Sui, Bo Zou, Helmut Ehrenberg, Gang Chen, Fei Du

**Affiliations:** ^1^ Key Laboratory of Physics and Technology for Advanced Batteries (Ministry of Education) State Key Laboratory of Superhard Materials College of Physics Jilin University Changchun 130012 China; ^2^ State Key Laboratory of Superhard Materials Jilin University Changchun 130012 China; ^3^ Institute for Applied Materials (IAM) Karlsruhe Institute of Technology (KIT) D‐76344 Eggenstein‐Leopoldshafen Germany

**Keywords:** aqueous binders, full cells, high‐performance materials, NASICON‐structured cathodes, sodium‐ion batteries

## Abstract

Polyanionic Na_3_V_2_(PO_4_)_2_F_3_ with a NASICON‐type structure is heralded as a promising cathode material for sodium‐ion batteries due to its fast ionic conduction, high working voltage, and favorable structural stability. However, a number of challenging issues remain regarding its rate capability and cycle life, which must be addressed to enable greater application compatibility. Here, a facile and effective approach that can be used to overcome these disadvantages by introducing an aqueous carboxymethyl cellulose (CMC) binder is reported. The resulting conductive network serves to accelerate the diffusion of Na^+^ ions across the interface as well as in the bulk. The strong binding force of the CMC and stable solid permeable interface protect the electrode from degradation, leading to an excellent capacity of 75 mA h g^−1^ at an ultrahigh rate of 70 C (1 C = 128 mA g^−1^) and a long lifespan of 3500 cycles at 30 C while sustaining 79% of the initial capacity value. A full cell based on this electrode material delivers an impressive energy density as high as 216 W h kg^−1^, indicating the potential for application of this straightforward and cost‐effective route for the future development of advanced battery technologies.

## 1 Introduction

Energy storage plays an important role in enabling a decarbonized society by storing electricity from sustainable sources such as wind and solar power. The past two decades have witnessed considerable technological advancements for rechargeable lithium‐ion batteries (LIBs), which are currently the storage technology of choice for portable electronic devices and electric vehicles; however, cost and safety issues remain two major obstacles that limit the use of LIBs in the smart grid. Taking into account the raw material abundance and battery cost as well as the similar electrochemical storage mechanism, room‐temperature sodium‐ion batteries (SIBs) based on Na‐ion shuttling between positive and negative electrodes are generally regarded as a promising choice for grid storage.[[qv: 1a–d]] Nevertheless, SIB technology must be further explored to obtain batteries that are more economical, safer, and show a high‐rate capability and longer durable life.[[qv: 2a–c]]

The robust 3D crystal structure and rapid Na‐ion insertion/extraction reaction in NASICON‐type phosphates afford a family of favorable electrode materials for SIB applications.[[qv: 3a–e]] Among the existing NASICON‐type materials, Na_3_V_2_(PO_4_)_2_F_3_ (NVPF) is of particular interest because of its high theoretical energy density of nearly 500 Wh kg^−1^, approaching that of LiFePO_4_ used in LIBs.^4^ However, such fluorophosphates usually suffer from an intrinsically poor electronic conductivity; thus, the advantage of the superionic conduction is offset.^5^ Many strategies have been attempted to tackle this issue, such as morphological engineering and surface modification by various carbonaceous materials including amorphous carbon, graphene, carbon nanotubes (CNTs), and CMK‐3.[[qv: 6a–e]] Unfortunately, these methods are usually time‐consuming, energy intensive, and costly.[[qv: 7a,b]] More importantly, the introduction of excess carbon can lower the energy density of the battery and increase the difficulty of binder adhesion. In addition, the cathode suffers from volume expansion, electrical contact loss, and separation from current collectors, especially at high current rates, leading to a short cycle life.

Though numerous efforts have been dedicated to the development of active materials, comparatively little attention has been paid to the optimization of binders. In fact, as one of the most important and indispensable components of a battery, the binder plays a crucial role in realizing a high‐rate capability and long‐term cycle life, especially for the negative electrodes.[[qv: 8a,b]] Up to now, poly(vinylidene difluoride) (PVDF) has been the most widely used binder; however, PVDF is usually dissolved in volatile, flammable, and explosive *N*‐methyl‐2‐pyrrolidone (NMP), which calls for a low processing humidity and poses a serious pollution threat to the environment. In addition to its insulating property, PVDF suffers from swelling and softening in ester‐based electrolytes due to the interactions between the polymer and electrolyte.^9^ In contrast, aqueous binders are receiving increased attention due to their low cost, nontoxicity, short drying time, nonrigorous processing humidity, and high conductivity. Sodium carboxymethyl cellulose (CMC), polyacrylic acid, and sodium alginate have all been widely applied as binders for the anode materials in SIBs.[[qv: 10a–c]] The enhancement in electrode performance based on the use of an aqueous binder can be understood in terms of the possibility for strong hydrogen bond formation between the hydroxyl functional group and active material, leading to stronger cohesiveness. Recently, CMC has been used as a binder for cathode materials;[[qv: 11a,b]] however, many open questions remain unsolved related to the role of aqueous binders in the improvement of sodium storage performance. Therefore, investigations focused on identifying the structure–performance relationship between the binder and the electrochemical characteristics are essential and highly desirable.

Herein, we demonstrate a facile and effective method for improving the rate capability and long‐term cycling stability of a NASICON‐structured cathode material utilizing CMC as a binder. By using this binder, we determine that the NVPF cathode can be used to realize a remarkable charging capability of 75 mA h g^−1^ at 70 C (1 C = 128 mA g^−1^) and a long lifespan of 3500 cycles at 30 C with a capacity retention of 79%. Further investigation shows that CMC is beneficial to the formation of a conductive network, protecting the structural and electrical integrity of the electrode as well as ameliorating the surface/interface property between the electrode and electrolyte.

## 2 Results and Discussion

### 2.1 Physical Properties and Architecture of the Electrode

The phase purity of pristine NVPF was first examined by powder X‐ray diffraction followed by a Rietveld refinement analysis, as shown in Figure S1 (Supporting Information). The data show sharp and well‐defined reflections that suggest a highly crystalline sample with no detectable impurities. The refined lattice parameters are *a* = *b* = 9.04 Å and *c* = 10.75 Å, consistent with previous reported values.^4^ Scanning electron microscopy (SEM) and transmission electron microscopy (TEM) were also used to characterize the sample morphology. NVPF nanoparticles ≈150 nm in size were found wrapped inside the amorphous carbon. In addition, the surface of the NVPF particles was coated by a thin layer of carbon with a thickness of ≈8.1 nm, which is beneficial for electronic transport between particles. The carbon content was quantified and found to be 4.2 wt% from the elemental analysis (C/H/N). High‐resolution TEM for a single crystalline particle showed a lattice plane spacing of 0.537 nm, which is consistent with the spacing of the (002) planes of tetragonal NVPF, confirming once again the structure type of the pristine sample.

**Figure 1:**
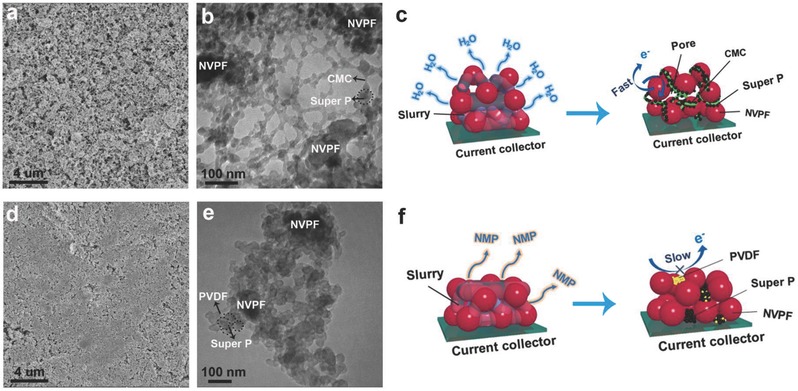
Morphology and architecture of the electrode. SEM and TEM images of the fresh electrodes. a,b) NVPF–CMC, d,e) NVPF–PVDF. c,f) Graphical illustration of the electrode drying process and the resulting electronic transport behavior for the NVPF–CMC and NVPF–PVDF electrodes, respectively.

In addition to the physical properties of the pristine materials, the composition and architecture of the working electrode can also affect the electrochemical performance, such as the rate capability and long‐term stability. Therefore, SEM and TEM were used to observe the morphologies of different NVPF electrodes prepared by mixing together the active material, conductive carbon, and CMC or PVDF binders (denoted NVPF–CMC or NVPF–PVDF, respectively). The NVPF–CMC electrode shows a loose and porous structure (**Figure** **1**a), which can favor electrolyte infiltration and the realization of sufficient electrochemically available interfaces and abundant ionic pathways;[[qv: 8a]] in contrast, the NVPF–PVDF electrode shows a more smooth and compact structure (Figure 1d). This is mainly due to the higher evaporation rate and shorter drying time of water during the electrode preparation process compared to NMP, as illustrated in Figure 1c,f. Further insight into the microstructure of the working electrode was gained from the TEM studies (Figure 1b and Figure S2 (Supporting Information)), which showed that the Super P and CMC chains build a conductive network that makes long‐term charge transfer easier. Isolated PVDF and Super P particles can limit the electrochemical performance at high rate, as shown in Figure 1c and Figure S3 (Supporting Information). Moreover, the stability of the cathode material with CMC was also confirmed, as shown in Figure S4 (Supporting Information).

### 2.2 High‐Rate Charge–Discharge Behavior

**Figure 2:**
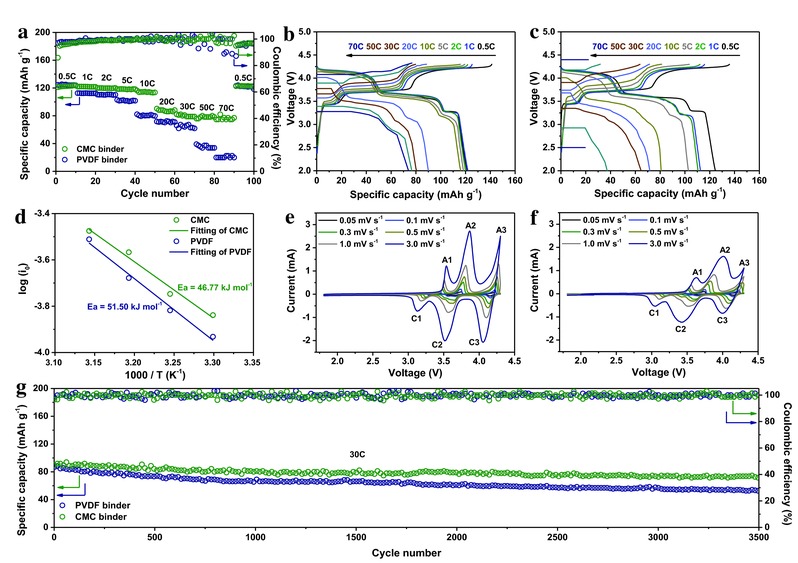
Electrochemical performance and kinetics analysis for the NVPF electrode. Discharge/charge profiles of a) NVPF–CMC and b) NVPF–PVDF electrodes measured at various rates. c) Rate performance of the two electrodes measured from 0.5 C to 70 C (1 C = 128 mA g^−1^). d) Calculation of the activation energy of the two electrodes. CV profiles of the e) NVPF–CMC and f) NVPF–PVDF electrodes recorded at sweep rates ranging from 0.05 to 3 mV s^−1^. g) Long‐term cyclability of the two electrodes at a rate of 30 C.

The galvanostatic charge–discharge profiles of NVPF–CMC and NVPF–PVDF were recorded in the voltage range between 2.0 and 4.3 V at a 0.5 C rate. As shown in Figure S4 (Supporting Information), three working plateaus were observed at ≈3.4, 3.7, and 4.2 V. The lower two plateaus can be attributed to the two‐step Na^+^ insertion reaction on the Na(2) site; the higher plateau is mainly due to the ion insertion/extraction of the second Na^+^ on the Na(1) site.^12^ Both the working electrodes show similar charge–discharge behavior and cycle stability at a low current density of 0.5 C regardless of the binder used (Figure S5, Supporting Information); however, the rate performance shows a strong dependence on the binder type. As shown in **Figure** **2**a–c, the rate performance of the NVPF@C cathode was evaluated at increased current rates from 0.5 C to 70 C for every 10 cycles. It can be seen that the cathode using CMC as the binder delivers a superior rate capability with no serious capacity loss from 20 to 70 C. The data show a high specific capacity of 75 mA h g^−1^ obtained at 70 C rate, which is almost 4 times higher than that of NVPF–PVDF. This is one of the highest rate performances reported for state‐of‐the‐art Na_3_V_2_(PO_4_)_2_F_3_ materials (see Table S1 in the Supporting Information). The corresponding discharge/charge profiles of NVPF–CMC show a long platform of 50 mA h g^−1^ at 3.25 V, which is in sharp contrast to the significantly polarized curves of the NVPF–PVDF electrode. These data clearly indicate that the CMC–Super P network greatly accelerates the charge/electron transfer and improves the rate capability.[[qv: 8a]] To gain further insight into the mechanism governing the sodium charge storage performance in the presence of the CMC binder, the electrochemical impedance spectra (EIS) were recorded at varying temperatures to calculate the activation energies for the working electrodes (Figure S6, Supporting Information). Based on the fits of the Arrhenius plots, the activation energies were calculated to be 46.7 and 51.5 kJ mol^−1^ for NVPF–CMC and NVPF–PVDF, respectively (Figure 2d). The lower activation energy for NVPF–CMC implies that the CMC binder facilitates faster transport of Na^+^ compared with that of the PVDF‐based electrodes.[[qv: 13a,b]] Furthermore, cyclic voltammetry (CV) were recorded at different scan rates from 0.05 to 3.0 mV s^−1^ to obtain the Na^+^ diffusion coefficient. As shown in Figure 2e**,**f, three pairs of symmetrical redox peaks were observed for both binder systems, which was consistent with the working plateaus observed in the charge–discharge profiles. It is worth noting that the redox polarization of NVPF–CMC is much lower than that of NVPF–PVDF, suggesting better electronic and ionic transport kinetics. The linear fit of the relationship between the peak currents and the square root of the scanning rates can be used to calculate the Na^+^ diffusion coefficient, *D*_Na_, on the basis of the Randles–Sevcik equation (Figures S7 and S8, Supporting Information).^14^ As listed in Table S2 (Supporting Information), the calculation of *D*_Na_ based on all the oxidation/reduction peaks observed for the NVPF–CMC electrode resulted in values that are larger than those calculated for NVPF–PVDF, which indicates the improved transportation of Na^+^ ions in the cathode and leads to the observed enhancement in the high‐rate charge–discharge capability.

To further verify the practical validity of the CMC binder in commercial SIBs, the weight ratio of the active materials, NVPF:Super P:binder, was increased from 7:2:1 to 9:0.5:0.5. It is important to note that although such a high weight ratio (active material:Super P:binder = 9:0.5:0.5) is similar to the value used for commercial LIBs based on LiFePO_4_ or LiCoO_2_ cathodes, it has not been utilized before for SIBs.[[qv: 15a,b]] Encouragingly, the NVPF–CMC electrode demonstrates an outstanding rate performance, i.e., a reversible capacity of 91 and 38 mA h g^−1^ at 10 C and 70 C, respectively (Figure S9, Supporting Information). These data highlight the potential for practical applications of the CMC binder in commercial SIBs. Moreover, the enhancement in the rate capacity using CMC as a binder was also confirmed for another NASICON‐type cathode, Na_3_V_2_(PO_4_)_3_/C (X‐ray diffraction (XRD) pattern in Figure S10 in the Supporting Information). As shown in Figures S11 and S12 (Supporting Information), the Na_3_V_2_(PO_4_)_3_/C cathode using a CMC binder delivers a significantly improved rate capability and cycle stability compared to that with PVDF as the binder, which further demonstrates the beneficial utilization of the CMC binder in SIBs.

### 2.3 Cycle Stability

**Figure 3:**
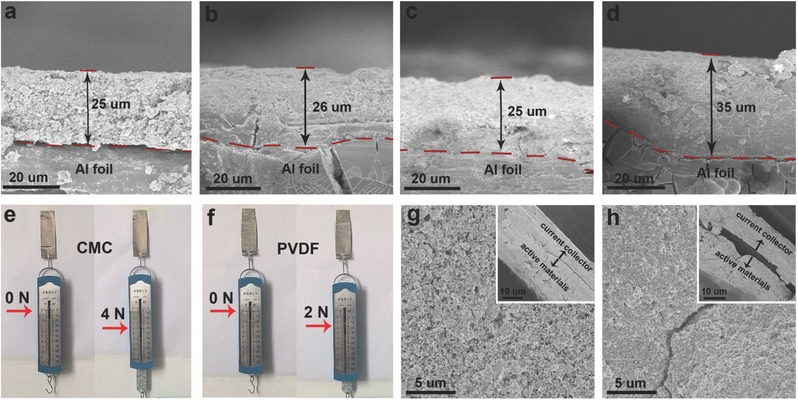
Binder stability and adhesive force. a,b) SEM images of the cross‐sections of the NVPF–CMC electrodes exposed to dry and wet conditions. c,d) SEM images of the cross‐sections of the NVPF–PVDF electrodes exposed to c) dry and d) wet conditions. Stretch test of the adhesive force for e) CMC and f) PVDF binders. SEM images of the g) NVPF–CMC and h) NVPF–PVDF electrodes after 100 cycles; the inset shows the corresponding cross‐sections of the electrodes.

**Figure 4:**
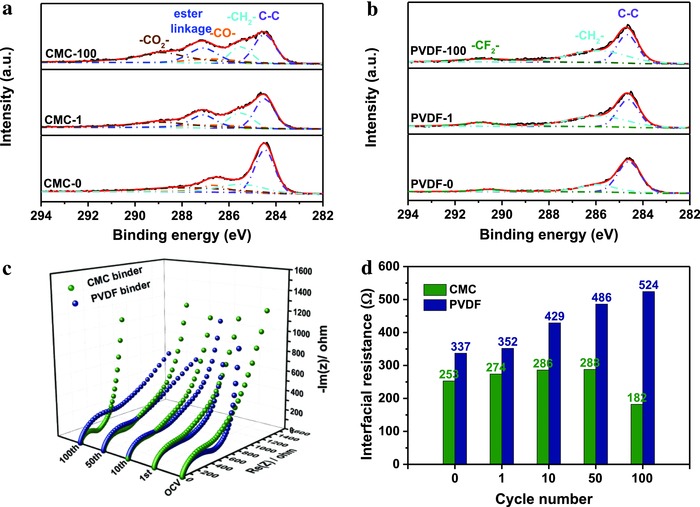
Component analysis for the SPI film and resistance evolution with cycling. XPS spectra showing the binding energies of C 1s measured for the a) NVPF–CMC and b) NVPF–PVDF electrodes following varying cycle numbers. c) Nyquist plots and d) calculated interfacial resistance of both the electrodes for selected cycles.

In terms of the conspicuously enhanced high‐rate capability, the long‐term durability of the two different working electrodes was also examined at 10 C (Figure S13, Supporting Information); the data show that the NVPF–CMC shows a higher capacity and better stability than NVPF–PVDF. The difference between the two electrodes increases once the current density is increased to 30 C. The NVPF–CMC electrode maintains 79% of its initial capacity following cycling, which is superior to the value of 61% for the NVPF–PVDF electrode (Figure 2g). The improved capacity retention observed after moving to the aqueous binder strongly suggests that although the binder only constitutes a small amount of the total mass of the working electrode, it plays a significant role in the realization of long‐term durability. Potential explanations for this enhanced durability can be understood as follows. (1) Swelling tests for the different binders were performed by wetting the electrodes in the electrolyte for the same amount of time. A cut section of the NVPF–CMC electrode was found to maintain its initial thickness of 25 µm following wetting with the electrolyte, which indicated negligible polymer/electrolyte interactions (**Figure** **3**a,b). In sharp contrast, the thickness of the NVPF–PVDF electrode clearly increased from 26 to 35 µm (Figure 3c**,**d). This thickness change can be attributed to a swelling effect due to the PVDF.^16^ (2) The adhesion forces of the different binders were also examined. A peeling experiment clearly showed that the force of adhesion for CMC was much stronger than for PVDF (Figure 3e**,**f and Videos S1 and S2 (Supporting Information)). Consequently, an ex situ SEM image of the NVPF–CMC electrode taken after 100 cycles showed perfect integrity, while the image for NVPF–PVDF clearly showed cracks that can be attributed to either the weaker adhesion force or degeneration of the PVDF (Figure 3g,h). 3. A solid permeable interface (SPI) is known to usually form on a cathode surface due to oxidation of the electrolyte, which can strongly affect the long‐term cycling stability.^17^ To further understand the effect of the binder on the SPI layer, an X‐ray photoelectron spectroscopy (XPS) analysis was carried out. As shown in **Figure** **4**b, the C 1s spectra of NVPF–PVDF show peaks at 286.0, 290.7, and 284.6 eV, which correspond to the carbon in the —CH_2_— and —CF_2_— bonds of PVDF and in the C—C bond of the carbon layer, respectively. In contrast, the binding energies for the NVPF–CMC electrode are found at 286.7, 288.6, and 285.4 eV, which are attributed to the —CO—, —CO_2_—, and —CH_2_— carbon atoms of the CMC binder, respectively (Figure 4a).^18^ After 10 cycles, the peak for the —CO— bond overlaps with another peak appearing at 287.1 eV and corresponding to the electrolyte decomposition products containing an ester linkage (—CO—O—).^19^ Although the contents of the oxidation products in the SPI film are different from that expected for SEI film formation on the anode side (usually RO—CO_2_—Na and R—CO_2_—Na[[qv: 20a,b]]), the surface film is supposed to prevent further electrolyte degradation and corrosion of the cathode materials.[[qv: 21a,b]] Indeed, the XPS data show that the SPI film is very stable after 100 cycles, without any obvious increase in content. Moreover, an SPI film can also ameliorate the kinetic property at the surface/interface between electrode and electrolyte. In Figure 4c, the EIS of both electrodes show a single semicircle in the high‐frequency region, indicating similar time constants for the SPI film resistance and charge‐transfer resistance (hereafter denoted as the interfacial resistance). As shown in Figure 4d, the interfacial resistance of NVPF–CMC is stable, and after 100 cycles, the interfacial resistance decreased from 253 to 182 Ω. In contrast, the interfacial resistance of NVPF–PVDF increased from 337 to 524 Ω. Thus, the SPI film induced by the CMC binder has a positive and efficient effect on the cycle stability.

### 2.4 High‐Performance Full Cell

**Figure 5:**
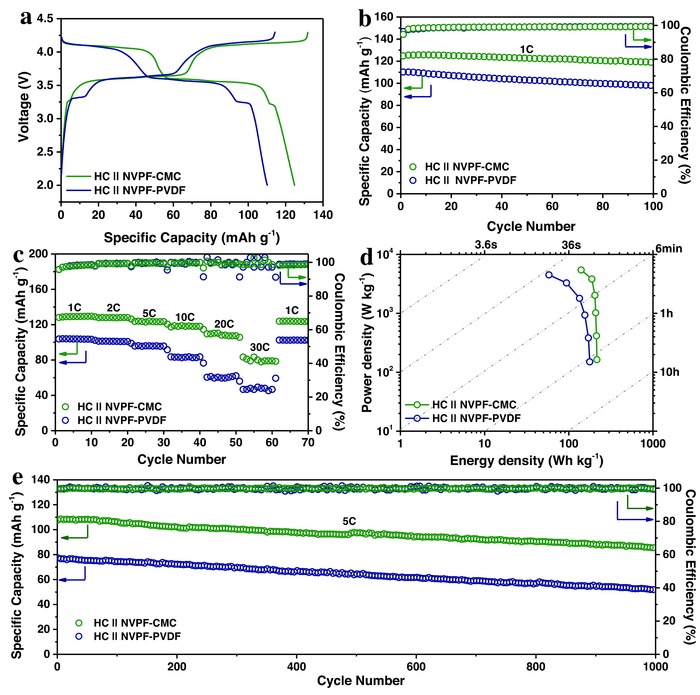
Electrochemical behavior of a high‐performance full cell. a) Typical charge–discharge profiles of the HC || NVPF–CMC and HC || NVPF–PVDF full cells measured at a current rate of 1 C. b) Cycling performance recorded over 100 cycles; voltage range is 2.0–4.3 V. c) Rate capability of the HC || NVPF–CMC and HC || NVPF–PVDF full cells. d) Ragone plots of the HC || NVPF–CMC full cell normalized to the total mass of the cathode and anode active materials. e) Long‐term cycling performance of the HC || NVPF–CMC and HC || NVPF–PVDF full cells at a high current rate of 5 C over 1000 cycles.

A full Na‐ion battery with a high energy density was constructed by combining an NVPF–CMC cathode with a hard carbon (HC) anode for operation in the voltage ranges of 2.2–3.9 V (Figure S14, Supporting Information) and 2.0–4.3 V (**Figure** **5**). To ensure full activation of the anode during the initial charging process, the mass loading was 1.2 times higher than that of the cathode. The NVPF–CMC || HC full cells in the optimized voltage range of 2.0–4.3 V show typical charge/discharge profiles with an average potential of ≈3.6 V, as shown in Figure 5a. These operating voltages are the same as those measured in the half cell for the NVPF–CMC cathode, which suggests that the low sodiation potential of HC (see Figure S15 in the Supporting Information) is close to the potential of Na metal and beneficial for realizing high‐energy density SIBs. The cycling performance and corresponding Coulombic efficiency were first tested at 1 C, as shown in Figure 5b; the data show no significant degradation in the voltage and capacity over 100 cycles. The full cell offers a 95.3% capacity retention after 100 desodiation and sodiation cycles, a compelling high voltage, outstanding cycle life, and a well‐defined voltage plateau, which are promising for the application of NVPF–CMC as a superior bipolar electrode for stationary batteries. The full cells were also tested at different rates of 1 C, 2 C, 5 C, 10 C, 20 C, and 30 C (Figure 5c). Even at a discharge rate of 30 C, the discharge capacity remained at 83.3 mA h g^−1^. The full cell can be used to deliver a large capacity of 124.8 mA h g^−1^ between 2.0 and 4.3 V with the energy density of the cell calculated to be ≈216 Wh kg^−1^ on the basis of the total cathode and anode mass, as shown in Figure 5d. To further study the long‐term cycling performance, the sodium full cell was cycled 1000 times at 5 C (Figure 5e). For the fast charging and discharging process, a good capacity retention of 79.4% was maintained even after 1000 cycles of sodium‐ion extraction and insertion.

## 3 Conclusion

We have demonstrated a facile and effective route to improve both the rate capability and cycle stability of NVPF by using an aqueous CMC binder. A conductive network consisting of CMC–Super P chains provides long‐range charge transfer pathways and a porous architecture. The electrochemical characterizations showed that Na‐ion diffusion in the bulk material and at the interface is accelerated due to improved electrochemical kinetics. Furthermore, the SPI interfacial film induced by the CMC binder limits the resistance increase and maintains the integrity of the electrode. Thus, the electrode enables an excellent capacity of 75 mA h g^−1^ at an ultrahigh rate of 70 C (1 C = 128 mA g^−1^) and a long lifespan of 3500 cycles at 30 C rate with capacity retention of 79%. Finally, a full battery cell assembly using hard carbon as the anode exhibits an energy density of 216 Wh kg^−1^, which suggests the potential for application in future scalable energy storage systems. The strategy we have proposed here is believed to be widely applicable to other cathode materials for sodium‐ion batteries.

## 4 Experimental Section

*Material Synthesis*: The Na_3_V_2_(PO_4_)_2_F_3_@C nanocomposite was prepared by a facile sol–gel method following the previous work.^4^ Typical preparation processes are described as follows: stoichiometric amounts of NH_4_VO_3_, NaF, and NH_4_H_2_PO_4_ (Sigma‐Aldrich, 99.9%) with a molar ratio of 2:3:2 were dissolved in deionized water. Then, a saturated citric acid [HOC(COOH)(CH_2_COOH)_2_] solution was added into the above solution until the ratio of vanadium:citric acid equaled 5:4. Next, the obtained solution was evaporated at 80 °C, dried at 120 °C for 12 h, and ground to form a precursor. Finally, the precursor was preheated at 300 °C for 4 h and sintered at 650 °C for 8 h under a nitrogen atmosphere with intermediate grinding to obtain the NVPF@C nanocomposite.

*Material Characterization*: The phase purity of the obtained samples was examined by XRD using a RigaKu D/max‐2550 diffractometer with Cu Kα source in the 2θ range of 10°–80° at a scanning rate of 1.7° min^−1^. Rietveld refinement was used to confirm the phase purity. The morphological features were observed by a Hitachi SU8020 scanning electron microscope and an FEI Tecnai G2 transmission electron microscope. Raman spectroscopy was carried out using a Renishaw in Via Raman system with an Ar‐ion laser excitation (λ = 514.5 nm). The samples for the swelling test were observed under a Hitachi SU8020 SEM. The NVPF–CMC and NVPF–PVDF electrodes were cut into two parts: one part was maintained in a dry condition and the other wetted in the electrolyte for the same time. Then, the thicknesses of the two electrodes for both the dry and wet conditions were measured by SEM of the cut section. The adhesion forces were measured by a peel‐test experiment. A piece of stainless‐steel foil was attached to another piece of stainless‐steel foil using either the CMC binder or PVDF binder in equal amounts; then, one of the stainless‐steel foils was fixed to a wall. Next, weights were added onto the free end of the stainless‐steel foil to draw it downward. The weights were added gradually and measured by a spring‐loaded thrust meter until the stainless‐steel foil completely peeled off from the other foil. Finally, the reading from the spring‐loaded thrust meter was taken. X‐ray photoelectron spectra were measured using a VG scientific ESCALAB‐250 spectrometer.

*Electrochemical Measurements*: All the electrochemical tests were carried out using coin‐type cells (CR2032). Sodium half cells were assembled using sodium foil as the counter electrode. The working electrodes were fabricated by coating a slurry prepared from a mixture of the active material, Super P conductive additive, and binder (sodium CMC dissolved in water or PVDF binder dissolved in NMP) in a weight ratio of 7:2:1, respectively, on an aluminum‐foil current collector. The electrode films were successively dried in a vacuum oven at 120 °C for 12 h. After patterning the electrode film into a square of area 0.8 × 0.8 cm^2^, the coin cells were assembled in a glove box. The loading mass of the materials was 1.1–1.6 mg cm^−2^. Glass fiber filters (Whatman GF/C) were used as separators for the cells. The electrolyte solution was prepared from 1 m NaClO_4_ dissolved in ethylene carbonate and propylene carbonate (1:1 v/v) with the addition of 5% fluoroethylene carbonate. Galvanostatic charge–discharge tests were carried out over a voltage range of 2.0–4.3 V at room temperature using a Land‐2001A (Wuhan, China) automatic battery tester. The specific capacities were calculated based on the Na_3_V_2_(PO_4_)_2_F_3_ mass. CV and electrochemical impedance spectra (EIS) were carried out using a VSP multichannel potentiostatic–galvanostatic system (Bio‐Logic SAS, France). CV curves were collected at different scanning rates ranging from 0.05 to 3.0 mV s^−1^. The EIS were measured in the frequency range from 1 MHz to 1 mHz. Sodium‐ion full cells were assembled in the same manner as the half cells except with hard carbon as the anode. Sodium‐ion full cells were cycled over different voltages ranging between 2.2–3.9 and 2.0–4.3 V.

## Conflict of Interest

The authors declare no conflict of interest.

## Supporting information

Supplementary

Supplementary

Supplementary
