# Supplementary material for: Moving to Aqueous Binder: A Valid Approach to Achieving High‐Rate Capability and Long‐Term Durability for Sodium‐Ion Battery
Source: Adv Sci (Weinh). 2018 Jan 20;5(4):1700768. doi: 10.1002/advs.201700768 (PMC5908374; doi:10.1002/advs.201700768)
Supplement: Supplementary file 1 — Supplementary [file ADVS-5-1700768-s002.pdf]

Copyright WILEY-VCH Verlag GmbH & Co. KGaA, 69469 Weinheim, Germany, 2018.

ADVANCED SCIENCE
Open Access

Supporting Information

for *Adv. Sci.,* DOI: 10.1002/advs.201700768

Moving to Aqueous Binder: A Valid Approach to Achieving High-Rate Capability and Long-Term Durability for Sodium-Ion Battery

*Jing Zhao, Xu Yang, Ye Yao, Yu Gao, Yongming Sui, Bo Zou, Helmut Ehrenberg, Gang Chen, and Fei Du**

Copyright WILEY-VCH Verlag GmbH & Co. KGaA, 69469 Weinheim, Germany, 2016.

Supporting Information

Moving to aqueous binder: a valid approach to achieving high-rate capability and long-term durability for sodium-ion battery

*Jing Zhao[1], Xu Yang[1], Ye Yao[1], Yu Gao[1], Yongming Sui[2], Bo Zou[2], Helmut Ehrenberg[2], Gang Chen[1] and Fei Du[1,]**

Supporting Information Figures

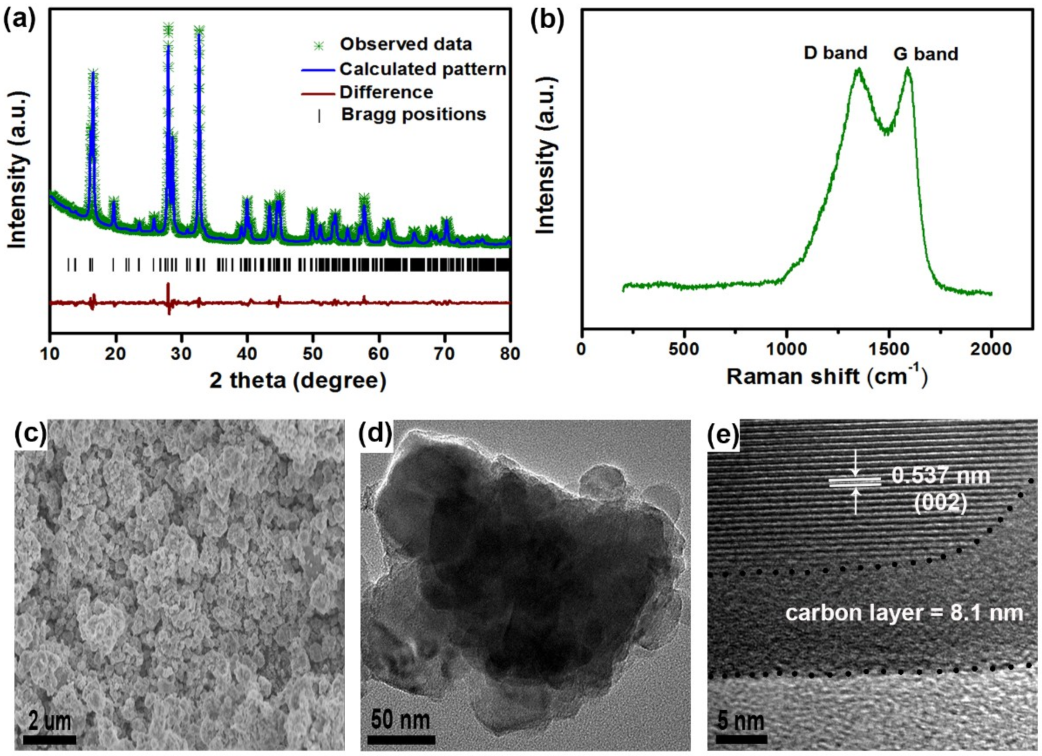

Figure S1 (a) Rietveld refinement based on the powder X-ray diffraction pattern of the NVPF@C nanocomposite; (b) Raman spectrum of the carbon in this nanocomposite; Morphological analysis of the NVPF@C nanocomposite: (c) SEM image, (d) TEM image, and (e) HRTEM image.

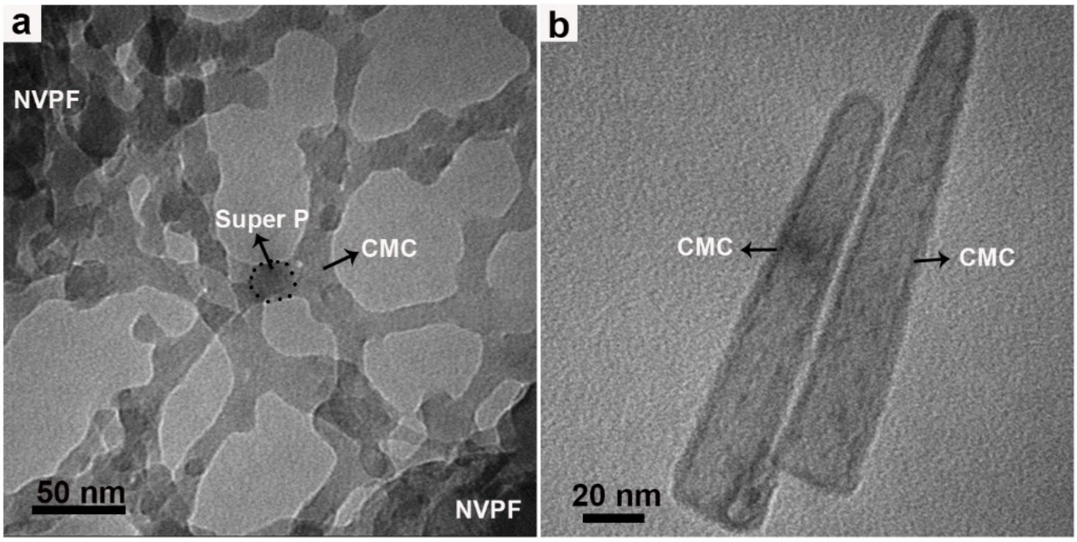

Figure S2 TEM images of (a) NVPF-CMC and (b) CMC powder.

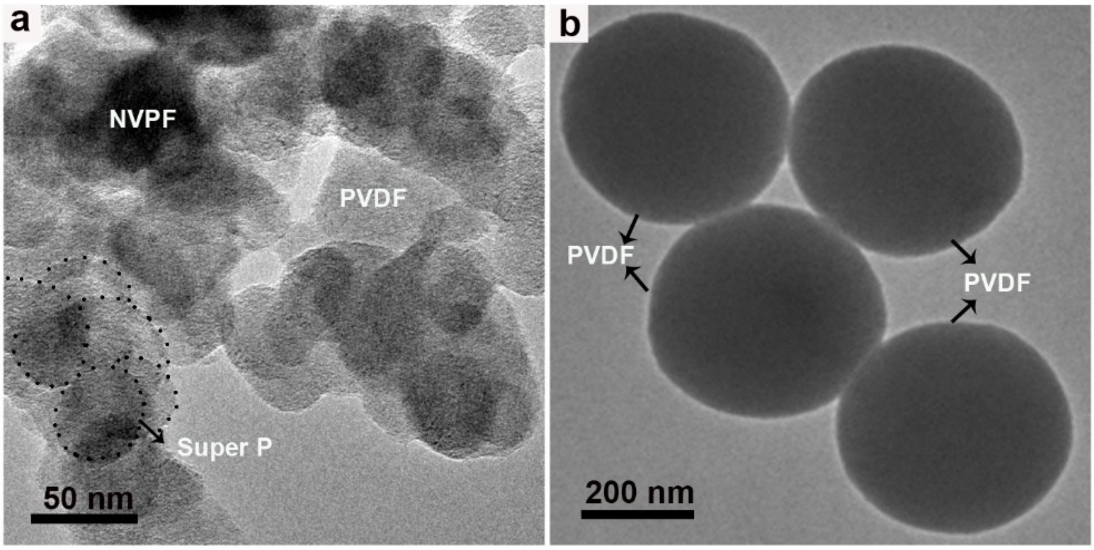

Figure S3 TEM images of (a) NVPF-PVDF and (b) PVDF powder.

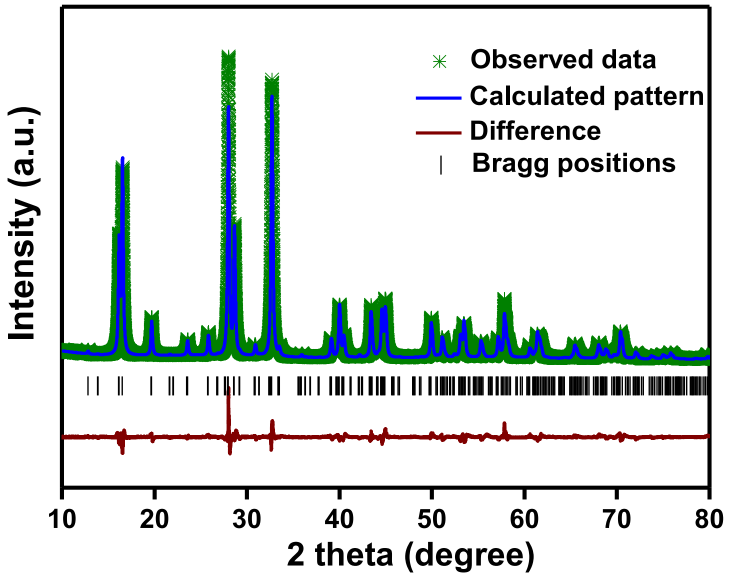

Figure S4 Rietveld refinement based on the powder X-ray diffraction pattern of the NVPF@C nanocomposite after being soaked with CMC and water for 1 day.

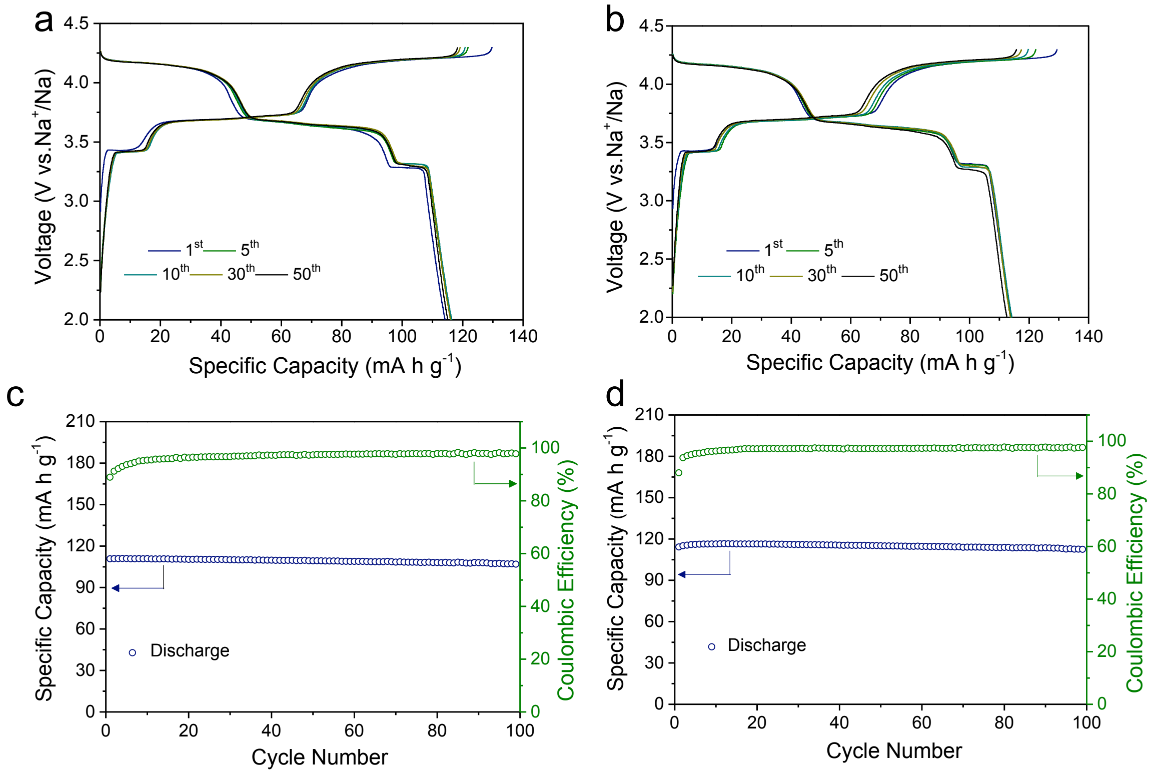

Figure S5 Charge-discharge profiles and cycle performance of (a,c) NVPF-CMC and (b,d) NVPF-PVDF electrodes at the current density of 0.5 C.

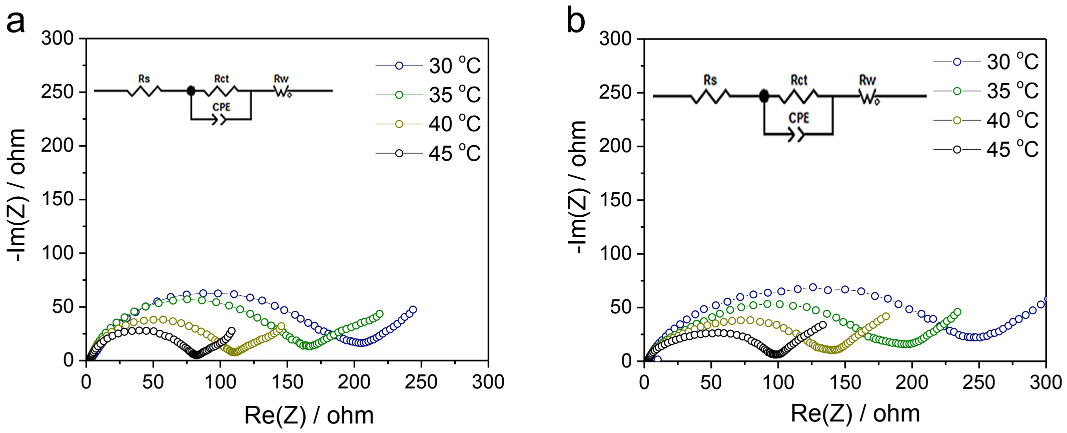

Figure S6 Nyquist plots of (a) NVPF-CMC and (b) NVPF-PVDF at an anodic potential of 3.6 V at different temperatures.

All the impedance profiles exhibit one compressed semicircle in the high-frequency region and a ~45° inclined line in the low-frequency range, attributable to the interfacial resistance (R_{ct}) and Warburg impedance (R_w), respectively. R_{ct} can be calculated using the equivalent circuit as shown in the inset of **Figure S6a and S6b**. The exchange current (i_0) and the apparent activation energy (E_a) for the sodium intercalated into NVPF can be calculated from Equation 1 and the Arrhenius equation (Equation 2):

$$i_0 = RT/nFR_{ct} \qquad (1)$$

$$i_0 = \mathrm{A}\, e^{-\frac{E_a}{RT}} \qquad (2)$$

where A is a temperature-independent coefficient, R is the gas constant, T is the absolute temperature, n is the number of transferred electrons, and F is the Faraday constant. **Figure 2d** shows the Arrhenius plots of log (i_0) as a function of 1000/T. The activation energies of NVPF electrodes using CMC and PVDF as binder are calculated to be 46.7 and 51.5 kJ mol^{-1}, respectively.

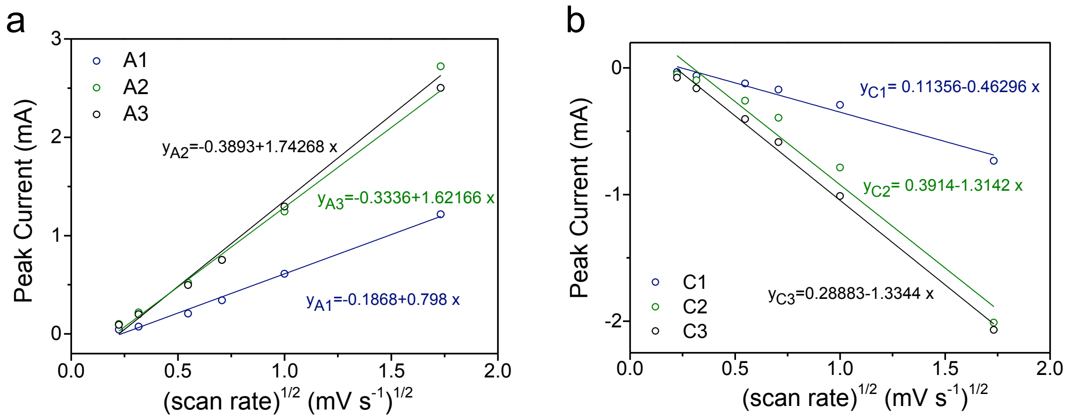

Figure S7 The relationship between the (a) anodic and (b) cathodic peak current (I_p) and the square root of the scan rate ($v^{1/2}$) for NVPF-CMC electrode.

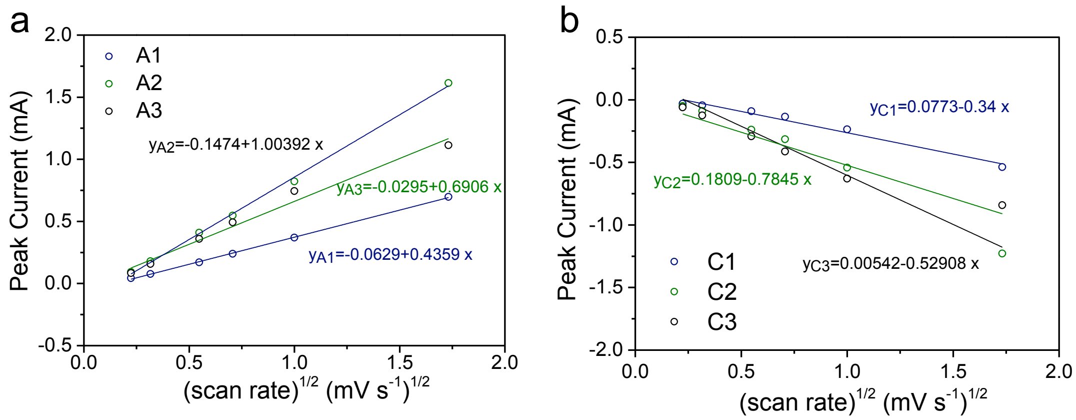

Figure S8 The relationship between the (a) anodic and (b) cathodic peak current (I_p) and the square root of the scan rate ($v^{1/2}$) for NVPF-PVDF electrode.

Na^+ diffusion coefficient D_{Na} can be calculated on the basis of Randles-Sevcik equation:

$$Ip=2.69\times10^5 n^{3/2} A D_{Na}^{1/2} C_{Na} v^{1/2}$$

where I_p is the peak current of anodic or cathodic peaks, n is the number of electrons per molecule during the intercalation, A is the effective contact area between the electrode and electrolyte, C_{Na} is the concentration of Na^+ in the electrode, and D_{Na} is the diffusion coefficient of Na^+ and v is the scan rate. **Figure S7 and S8** shows the linear relationship of I_p versus $v^{1/2}$ plots. Calculated D_{Na} as listed in Table S1.

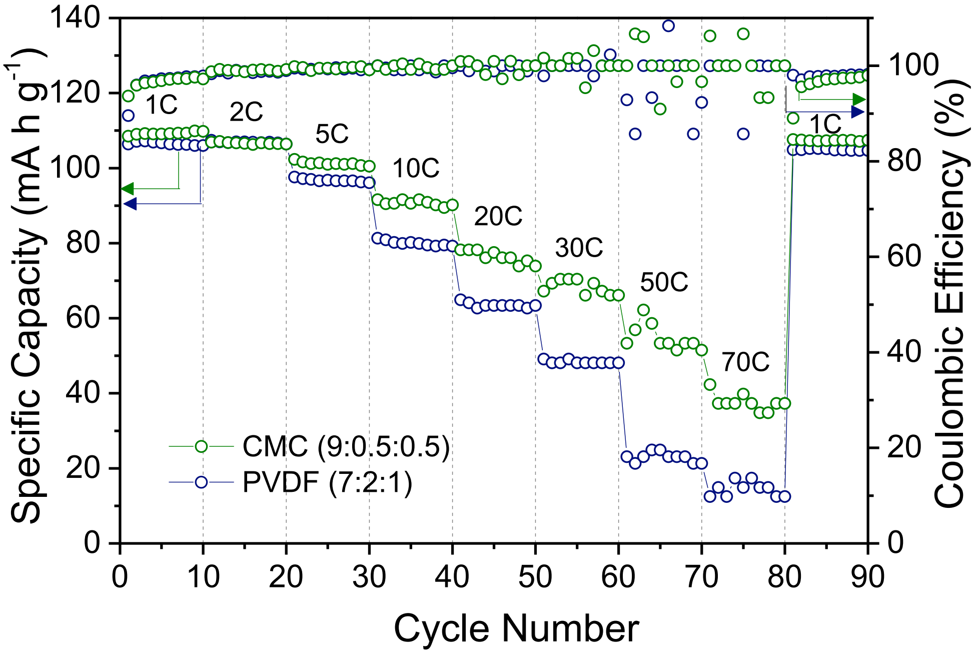

Figure S9 Rate performance of CMC (9:0.5:0.5) and PVDF (7:2:1).

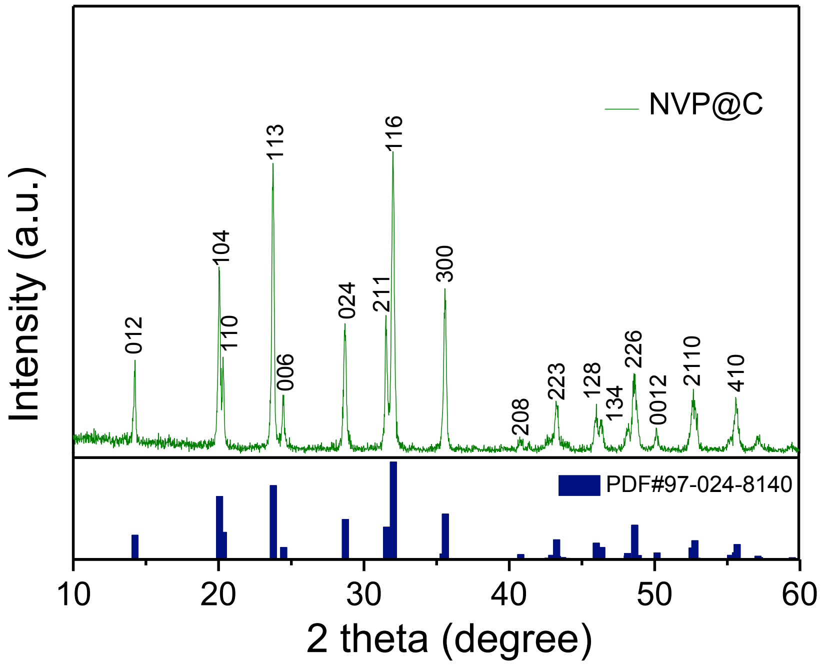

Figure S10 XRD pattern of $Na_3V_2(PO_4)_3/C$.

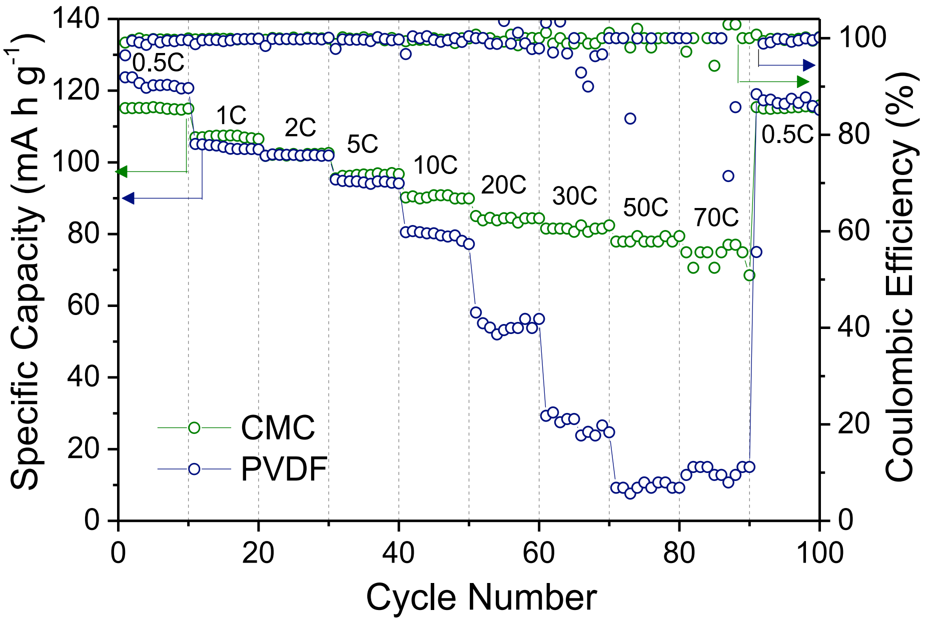

Figure S11 Rate performance of $Na_3V_2(PO_4)_3/C$ with CMC and PVDF binder, respectively.

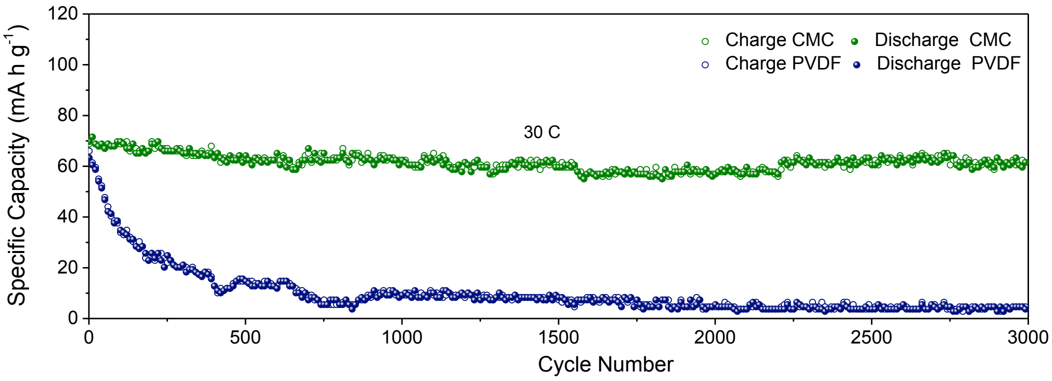

Figure S12 Long cycle performance of NVP-CMC and NVP-PVDF electrode at 30 C rate.

To further check the applicability of CMC binder in SIBs, another famous cathode, $Na_3V_2(PO_4)_3/C$ (NVP) is prepared and its sodium storage properties are comparatively studied in different binders. As shown in Fig. **S11**, the $Na_3V_2(PO_4)_3/C$ cathode using CMC as binder also demonstrates superior rate capability with discharge capacities of 85 and 75 mA h g^{-1} at the 20 and 70 C rates, respectively, superior to the performance using PVDF. Impressively, the capacity retention over 3000 cycles at 30 C rate is obtained as high as 91%, whereas, the cathode in PVDF hardly delivers any capacity after 1000 cycles. All these manifested outstanding performances demonstrate the significant value and prospect of CMC binder in the commercial applications of SIBs.

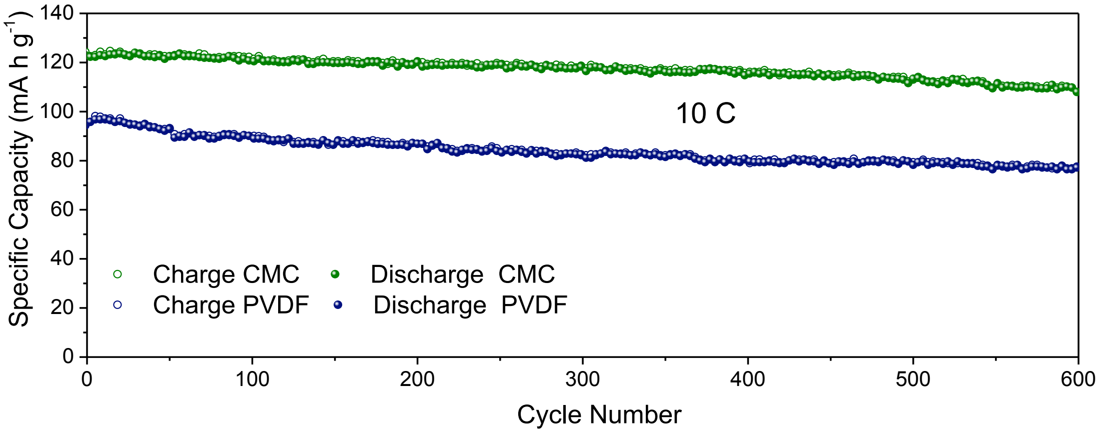

Figure S13 Long cycle performance of comparison of NVPF-CMC and NVPF-PVDF electrode at the current density of 10 C.

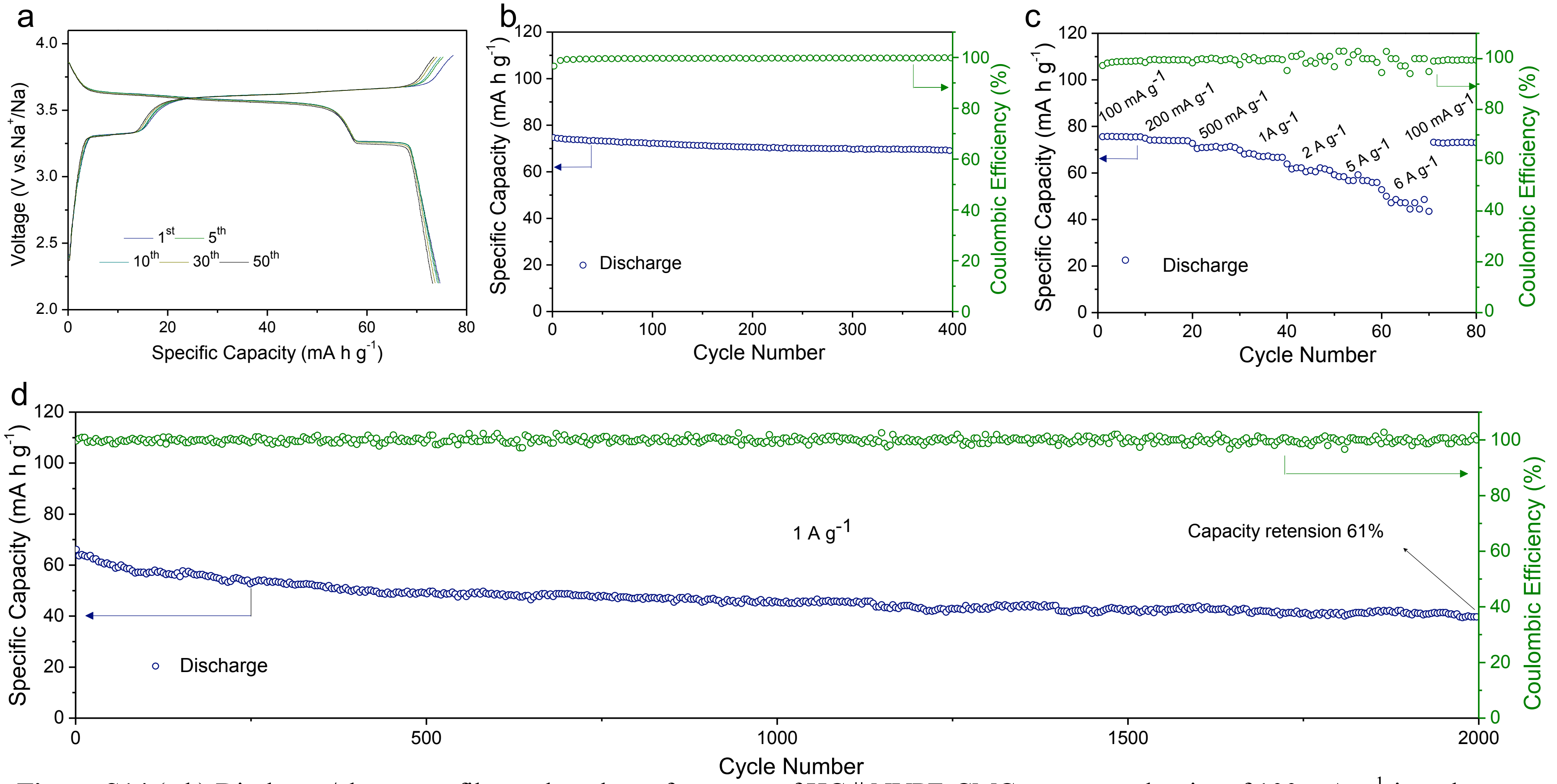

Figure S14 (a,b) Discharge/charge profiles and cycle performance of HC || NVPF-CMC at current density of 100 mA g^{-1} in voltage range of 2.2 - 3.9 V. (c) Rate performance of HC || NVPF-CMC from 100 mA g^{-1} to 6 A g^{-1}. (d) Long-term cycling of HC || NVPF-CMC at current density of 1 A g^{-1}.

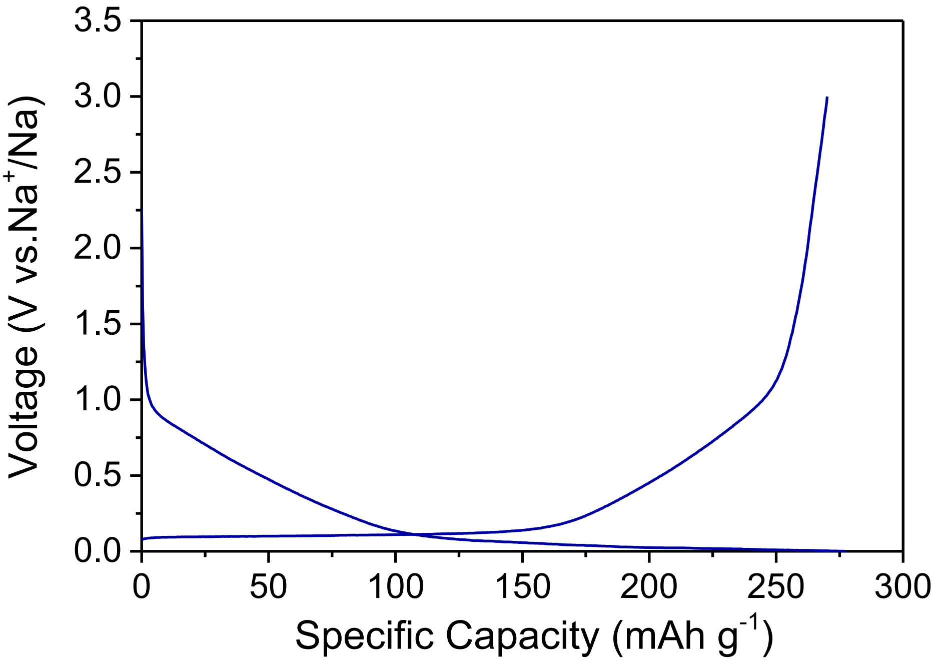

Figure S15 Discharge/charge profiles of hard carbon.

Surppoting Information Tables

Table S1 Performance comparision between our work and other polyanionic $Na_3V_2(PO_4)_2F_3$ and its similar products in literature.

Material	Rate Performance	Cycle Life	Ref.
$Na_3V_2O_2(PO_4)_2F$/ Ru-doped	45 mA h g^{-1} at 100 C	7500 cycles at 20 C with 55 mA h g^{-1}	1
$Na_3V_2O_2(PO_4)_2F$/ RuO_2 coated	71 mA h g^{-1} at 40 C	1000 cycles at 20 C with 95 mA h g^{-1}	2
$Na_3V_2O_2(PO_4)_2F$/ Graphene	79 mA h g^{-1} at 10 C	40 cycles at 1 C with 112 mA h g^{-1}	3
$Na_3V_2(PO_4)_2F_3$@C @ CMK-3	63 mA h g^{-1} at 30 C	5000 cycles at 50 C with 65 mA h g^{-1}	4
$Na_3V_2(PO_4)_2F_3$@C	57 mA h g^{-1} at 30 C	3000 cycles at 30 C with 40 mA h g^{-1}	5
$Na_3V_2(PO_4)_2F_3$/ Nanoflowers	32 mA h g^{-1} at 10 C	500 cycles at 0.1 C with 99 mA h g^{-1}	6
$Na_3V_2O(PO_4)_2F_2$/ Graphene	77 mA h g^{-1} at 50 C	1000 cycles at 50 C with 51 mA h g^{-1}	7
$Na_3V_2O_{2x}(PO_4)_2F_{3-2x}$ /rGO	72 mA h g^{-1} at 20 C	250 cycles at 0.1 C with 108 mA h g^{-1}	8
$Na_3V_2O_{2x}(PO_4)_2F_{3-2x}$ /MWCNT	41 mA h g^{-1} at 20 C	100 cycles at 0.1 C with 102 mA h g^{-1}	9
$Na_3V_2(PO_4)_2F_3$/CMC	75 mA h g^{-1} at 70 C	3500 cycles at 30 C with 70 mA h g^{-1}	This work

Table S2 The diffusion coefficient of Na^+ of NVPF-CMC and NVPF-PVDF electrode.

D_{Na} (cm^2S^{-1})	A1	A2	A3	C1	C2	C3
CMC	2.5×10^{-11}	2.6×10^{-10}	8.0×10^{-10}	8.5×10^{-12}	1.5×10^{-10}	5.4×10^{-10}
PVDF	7.5×10^{-12}	8.6×10^{-11}	1.5×10^{-10}	4.5×10^{-12}	5.3×10^{-11}	8.5×10^{-11}

Supplementary Reference

[S1] M. Peng, D. Zhang, L. Zheng, X. Wang, Y. Lin, D. Xia, Y. Sun, G. Guo, *Nano Energy* **2017,** *31*, 64-73.

[S2] M. Peng, B. Li, H. Yan, D. Zhang, X. Wang, D. Xia, G. Guo, *Angew. Chem. Int. Ed.* **2015,** *54* , 6452-6456.

[S3] H. Jin, J. Dong, E. Uchaker, Q. Zhang, X. Zhou, S. Hou, J. Li, G. Cao, *J. Mater. Chem. A* **2015,** *3*, 17563-17568.

[S4] Q. Liu, X. Meng, Z. Wei, D. Wang, Y. Gao, Y. Wei, F. Du, G. Chen, *ACS Appl. Mater. Interfaces* **2016,** *8*, 31709.

[S5] Q. Liu, D. Wang, X. Yang, N. Chen, C. Wang, X. Bie, Y. Wei, G. Chen, F. Du, *J. Mater. Chem. A* **2015,** *3*, 21478.

[S6] Y. Qi, L. Mu, J. Zhao, Y.-S. Hu, H. Liu, S. Dai, *J. Mater. Chem. A* **2016,** *4* , 7178-7184.

[S7] X. Xiang, Q. Lu, M. Han, J. Chen, *Chem. Commun.* **2016,** *52*, 3653-3656.

[S8] P. R. Kumar, Y. H. Jung, S. A. Ahad, D. K. Kim, *RSC Adv.* 2017, 7, 21820–21826.

[S9] P. R. Kumar, Y. H. Jung, C. H. Lim, D. K. Kim, *J. Mater. Chem. A* **2015,** *3*, 6271-6275.
